# Quantitative Consensus in Systematic Reviews: Current and Future Challenges in Translational Science

**DOI:** 10.6026/97320630014086

**Published:** 2018-02-28

**Authors:** Francesco Chiappelli, Vandan R. Kasar, Nicole Balenton, Allen Khakshooy

**Affiliations:** 1 Laboratory of Human Psychoneuroendocrine-Osteoimmunology; School of Dentistry, UCLA Center for the Health Sciences, Los Angeles, CA 90095-1668; 2 Evidence-Based Decision Practice-Based Research Network, DGSO, Los Angeles, CA 91403; 3 Department of the Health Sciences, CSUN, Northridge, CA 91330; 4 Dental Group of Sherman Oaks, Los Angeles CA 91403; 5 School of Dentistry, UCSF Center for the Health Sciences, San Francisco, CA 94143; 6 Rappaport Faculty of Medicine, Technion-Israel Institute of Technology, Haifa, Israel 3109601

**Keywords:** Translational science, research synthesis, PICOTS question, complex systematic reviews, meta-analysis, Bayesian statistics, additive meta-analysis

## Abstract

Translational science conceptualizes healthcare as a concerted set of processes that integrate research findings from the bench to the bedside. This model of healthcare is effectiveness-focused, patient-centered, and evidence-based, and yields evidence-based revisions of practice-based guidelines, which emerge from research synthesis protocols in comparative effectiveness research that are disseminated in systematic reviews. Systematic reviews produce qualitative and quantitative consensi of the best available evidence. The quantitative consensus is derived from meta-analysis protocols that are often achieved by probabilistic approach Bayesian statistical models.

## Consensus of the Best Evidence Base in Science

Translational science in healthcare is articulated along three phases of translation (T) of research findings from the bench to the bedside [1]: In T1, laboratory studies define and characterize the genomic, proteomic and interactomic biology and physiopathology that afflict the patient. In T2, clinical research documents the efficacy of certain interventions and tests their effectiveness, in a comparative effectiveness research paradigm, specifically for cost/benefit ratio, and risk/benefit ratio at the individual patient level; and in T3, the optimal modalities for healthcare delivery, stakeholder engagement, and preventive services are designed and implemented.

Contemporaneously, the Institute of Medicine defined comparative effectiveness research as *"... the generation and synthesis of evidence that compares the benefits and harms of alternative methods to prevent, diagnose, treat, and monitor or improve the delivery of care. The purpose of (comparative effectiveness research) is to assist consumers, clinicians, purchasers, and policy makers to make informed decisions that will improve health care at both the individual and population levels..."* [2].

Taken together, comparative effectiveness research is that specific component of translational science that specifically refers to the concerted effort of developing, examining, testing and comparing the effectiveness (i.e., effectiveness-focused) of different treatment interventions targeted to a well-characterized condition in individual patients (i.e., patient-centered), and based on the best available evidence (i.e., evidence-based). Comparative effectiveness research yields new and improved evidence-based revisions of practice-based guidelines in healthcare [4, [5, 6, 7, 8, 9, 10, 11, 12, 13]. Comparative effectiveness research directly compares existing health care interventions and modalities to determine which work best for which patients and under which specific circumstance. It seeks to obtain the qualitative and quantitative consensus of high level and high-quality peer-reviewed research evidence that specifically documents which intervention or modality poses the greatest benefits and harms for its relative cost.

In short, the goal of comparative effectiveness research is to proffer systematic reviews of the literature pertinent to a given bibliome for the relationship between certain variables, including treatment or exposure and effectiveness of clinical outcome. Systematic reviews of the pertinent literature with the research synthesis design yield qualitative and quantitative consensus statements, which are the foundational elements of effectiveness-focused, patient-centered and evidence-based translational science in healthcare for improving the health of individuals, families and society [4, [5, 6, 7, 8, 9, 10, 11, 12, 13].

Translational science is at the core of modern contemporary patient-centered healthcare, and is grounded in the systematic process of comparative effectiveness research and evidence-based recommendations for clinical guidelines just outlined. It requires stringent and systematic research synthesis of peer-reviewed research reports searched across several databases based on their adherence to the patient description (P) - intervention description (I) - comparators description (C) - outcomes description (O) - timeline (T) - and settings (S) (PICOTS question). Upon scrutiny of the PICOTS question by means of the analytical framework, key questions, and inclusion and exclusion criteria, the resulting literature search is refined for its specific correspondence to PICOTS, yielding the bibliome [7]. Each report in the bibliome is evaluated for research level [7, 9] and quality (e.g., GRADE, 14; Risk of Bias, 15), and analyzed for acceptable sampling [7, 9]. A compexio similitudinis, a consensus of the evidence converging to the best available evidence in the bibliome under study is generated based on the qualitative findings, which can be quantified as well [16], and by a meta-analysis of the quantitative data [7, 9]. Meta-analysis is a statistical analysis protocol, generally run in the probabilistic paradigm [7, 9, 18]. It was originally proposed by Karl Pearson [19, 20]in his report of enteric fever inoculation statistics. When comparing infection and mortality among soldiers who had volunteered for inoculation against typhoid fever with the mortality of other soldiers who had not volunteered for such an inoculation across the British Empire, Pearson re-grouped the study observations into larger groups to increase sample sizes, and combined the results from multiple studies to raise the sample sizes of individual groups. This process yielded increased statistical power over the individual studies. His approach also resolved uncertainty, improved estimates of the effect size, and produced a weighted average of the study results [19, 20].

## Meta-Analysis for Quantitative Consensus in Research Synthesis

Meta-analysis proffers the ability to perform a statistical analysis by combining several studies, and produces the quantitative consensus across these studies, without predicting the results of a future larger study. It, like other parametric biostatistical tests [7, 9, 17], rests on several assumptions [21-23].

Meta-analysis proffers a quantitative weighted average of the effect size of an intervention, a degree of association between a risk factor and a disease, or a value of accuracy of a diagnostic test. It reveals the consensus of relationships across multiple nonheterogeneous studies [7, 23].

Meta-analysis integrates the outcomes across several studies, so long as they are measurements of the same outcome variable - that is to say, homogeneous. Homogeneity is visualized by L'Abbé plots, which identify the studies with different results from other studies, and the study arms responsible for such differences. L'Abbé plots are visual aids that do not account for sampling error, and may therefore be misleading [24].

Statistical heterogeneity [7, 23, 25] is established statistically by means of the Cochran Q statistics, or the I2 statistics. Cochran Q is a block matched X2 read on the χ2 distribution with (k - 1) degrees of freedom, computed by summing the squared deviations of each study's estimate from the overall meta-analytic estimate. It weighs each study's contribution equally but has relatively low power in establishing that k treatments are statistically different.

I2 tests the degree of inconsistency among studies as the percentage of total variation due to heterogeneity rather than chance, such that I2 > 50% reflect moderate to high heterogeneity. It is rendered as I2 = 100%x(Q - df)/Q Where Q is Cochran's heterogeneity statistic and df is the degrees of freedom.

Heterogeneity examines the fundamental question of what is pulled together, what is used to produce overall inferences: in short, what sets of studies are 'combinable' [7, 23]. If heterogeneity is low-to-moderate, or absent, then the analysis can safely employ either the fixed-effect or the random-effect models of meta-analytical inference.

The fixed-effect model [7, 18, 23] assumes that the size of the treatment effect is homogeneous and fixed across all studies, and that the variation seen between studies is due to random events. It provides a weighted average of the study estimates, the weights being the inverse of the variance of the study estimate. Larger studies with larger weights dominate the outcome of a fixed-effect model meta-analysis, because they "drag" the meta analysis' fixed point toward their own finding. The relevance of smaller studies with smaller weights can be diminished to the point of being all but ignored. This bias is particularly grave when there is some degree of heterogeneity because the inference is dragged by the larger study, irrespective of intrinsic heterogeneity.

By contrast, the random-effect model [7, 18, 26] assumes that the treatment effects are heterogeneous across the studies. It tends to increase the variance of the summary measure, and loses some degree of stringency compared to fixed-effects models. Random effect inferences yield a weighted average of the effect sizes across studies, but here the weight results from inverse variance weighting obtained by aggregating two or more random variables to minimize variance: each random variable in the sum is weighted in inverse proportion to its variance. The weighted average of the effect sizes can also be obtained by un-weighting of the inverse variance weighting, which requires applying a random effects variance component that is derived from the extent of variability of the effect sizes of the underlying studies. The greater the heterogeneity in effect sizes, the larger the required un-weighting.

To be sure, when heterogeneity is extreme or excessive, then random-effect meta-analysis inferences tend toward the unweighted average effect size across the studies. At the other extreme, when heterogeneity is minimal, effect sizes are similar, and variability does not exceed sampling error, then no random effects variance component can be obtained: the random effects meta-analysis defaults de facto to a fixed effect meta-analysis with only inverse variance weighting. In brief, the extent of unweighing depends on the heterogeneity of precision of measurement, or on the heterogeneity of effect size.

Heterogeneity results more likely from systematic error and random error, than underlying true differences in study effects. The presumption that a larger variability in study sizes or effect sizes points to flagrant faults in larger studies but not in smaller studies is, in this model, groundless and fallacious. The redistribution of weights under this model bears no relationship to the contribution of each individual study [7, 18].

The most widely used method to estimate heterogeneity is the Der Simonian-Laird Q-based, maximum likelihood, profile likelihood approach. It incorporates the heterogeneity of effects across the studies in the analysis of the overall treatment efficacy, and can be extended to include relevant covariates to reduce the heterogeneity and allow for more specific therapeutic recommendations. The Kontopantelis-Reeves restricted maximum likelihood approach [7, 23, 27] is also used.

To be clear, the use of the random-effect model in calculating confidence intervals, for instance, results in wider intervals and a more conservative estimate of treatment effect, compared to the fixed-effect. Thus, and to reconcile these caveats, sensitivity analysis is required whereby meta-analyses are re-analyzed excluding un-blinded or open-label studies, using the Mantel- Haenszel fixed-effect inference model [7, 23]. By contrast, random-effect models are more appropriate than fixed-effects models when some degree of heterogeneity is detected. Random effects models are common in comparative effectiveness research, where variability often arises from experiment-related errors.

In part to resolve the controversy between fixed-effect and random-effect models, the quality-effect model follows a distinct approach of adjustment for inter-study variability by incorporating a relevant control variable, named component in this context. Components can include estimates of quality of the evidence (e.g., Risk of Bias assessment score) in each study. Components are integrated in the meta-analysis in addition to the weight based on the intra-study differences, as noted above. The quality-effect model corrects for the quality-adjusted weight of the ith study, and taui (Τi) is introduced as a composite score based on the quality of other studies. Quality-adjusted weights determine the redistribution, such that higher quality studies have greater weight in estimating overall effect size [7, 18].

Decreased power may follow segregation of the studies into subgroups based on the component criteria value. But, and in answer to *"...how many studies do you need to do a meta-analysis?", Valentine and collaborators (2010) respond "...given the need for a conclusion, the answer is "two studies," because all other synthesis techniques are less transparent and/or are less likely to be valid..."* [29]. Nonetheless, power analysis for meta-analysis should be established prospectively or retrospectively, but in is rarely considered, despite the availability of the specialized SAS® Macro and other related software programs [29, 30].

When heterogeneity is detected, its origin and cause can be explored by meta-regression [31]. The simplest type of meta regression uses summary data from each trial, such as the average effect size, average disease severity at baseline, and average length of follow-up to explore which types of patient specific factors or study design factors contribute to the heterogeneity. Meta-regression is a sine qua non preliminary step for meta-analysis despite its inherent limitation for identifying important factors, such as patient-specific features, related to the size of treatment effect. When heterogeneity is excessive, meta analysis is not warranted.

Bias and fallacy are as pervasive in meta-analysis as they are in primary data analysis [15, 32], and in both cases blunt inferential validity. Bias cannot be eliminated and arises from sampling error, measurement error, publication bias, and various other sources.

Publication bias is an unavoidable weakness of all bibliomes, and its effects upon the quantitative consensus in comparative effectiveness research cannot be ignored. It is quantified by the Begg rank correlation and the Egger weighted regression methods [33, 34]. A useful graphical representation of publication bias [33] is the funnel plot, in which the magnitude of the effect is plotted against the sample size. The true mean, m, is taken as 0, and the standard deviation as 1. The difference between two ideally equal groups that show significant and non-significant results should form a funnel-like shape that extends to infinity along the 95% confidence intervals. Funnel plot asymmetry is rendered as the Begg-Mazumdar statistics [35], a non-parametric rank correlation of intervention effect estimates and their sampling variances.

Several instruments [7, 23, 36, 37] have been developed and validated for assessing the quality of meta-analyses, principally from the viewpoint of inherent sources of bias, and inferential model, including the Preferred Reporting Items for Systematic Reviews and Meta-Analyses (PRISMA) Table 1 [37, 38, 39, 40]. PRISMA was developed as a simple binary checklist (yes/no), which limits its psychometric validation.

To circumvent this hurdle, we have revised PRISMA in a manner similar to our previously revision of the AMSTAR for assessing the quality of systematic reviews [41], and have obtained quantitative data on the reliability and the criterion validity of our Revised PRISMA. The R-PRISMA proffers a quantifiable means to assess the reporting transparency, sound research protocols, and bias. It yields a score that represents the quality of the systematic reviews and meta-analyses before their integration into secondary-level systematic reviews (Kasar et al, in preparation).

In brief, meta-analysis is an elaborate statistical protocol, which can be estimated by means of standard software (e.g., BioStat Comprehensive Meta-Analysis software version 1.0.25), and whose inferential stringency largely depends on the homogeneity and quality of the individual studies.

## Complex Systematic Reviews and Cumulative Meta-Analysis

In translational science, and specifically in comparative effectiveness research, similar PICOTS may overlap for any given bibliome, and produce related systematic reviews. It is increasingly common that any given PICOTS include several existing systematic reviews within its bibliome. It follows that a novel trend of comparative effectiveness research encompasses a secondary-level systematic review in which both the qualitative and quantitative consensi are obtained from primary research and from primary-level systematic reviews.

To be clear, the quality of research must be determined, for example by the Risk of Bias instrument, when generating a primary level systematic review. The quality of existing, primary level systematic reviews must also be established (e.g., RAMSTAR, [41]; QUORUM, [37]; PRISMA, [38, 39, 40]) before including them into secondary-level systematic reviews, which are also referred to as systematic reviews of systematic reviews.

The term 'complex' systematic reviews have been used to describe systematic reviews of systematic reviews, which we refer to here as secondary-level systematic reviews. The term 'complex' should optimally be reserved for the situation where the investigator finds both new primary research not heretofore synthesized as a systematic review within the bibliome, and existing primary-level systematic reviews. The former will have to be processed as a novel independent systematic review, and the latter will require a secondary-level systematic review of the existing systematic reviews.

The third step in the process will juxtapose the two independent sets of consensi generated by the two independent operations mentioned above, into one comprehensive revised and updated consensus. The terminology 'complex' systematic review is more appropriate to this elaborate three-step process (i.e., tertiary-level systematic review) than the rather simple secondary-level systematic review of systematic reviews.

In either secondary-level or a tertiary-level systematic review, an important question pertains to how the consensus of the best available evidence is generated. The qualitative consensus may only require updated statements. If quantifications have been obtained [17], then a simple quantitative updating is required.

By contrast, the generation of the quantitative consensus in secondary-level or a tertiary-level systematic review is problematic. The quantitative consensus in primary systematic reviews is produced by meta-analysis along the stringent criteria noted above. It has been proposed that a 'cumulative' meta-analysis may be the protocol of choice to produce the quantitative consensus across multiple existing systematic reviews, each presenting one or more meta-analyses.

Practically speaking, a cumulative meta-analysis simply keeps adding new entries to an existing meta-analysis. But, from the perspective of statistical theory, this approach is as flawed as adding new groups in an existing t test analysis. As it is statistically improper to add new groups to a t test analysis within a given experimental design targeted to answer an original research question because it artificially increases the risk for Type I and Type II error, so it is statistically improper to add new studies in an existing meta-analysis within a given research synthesis design targeted to answer an original PICOTS question. The following equation presents this relationship: p(Type I error)=1-(1-α)c Where c is the number of comparisons dictated by the number of groups in the study

The same equation applies when c is replaced by e, where e is the number of entries of independent meta-analyses generated in primary level systematic reviews now being pulled into a secondary-level or a tertiary-level systematic review.

In brief, as much as performing multiple t tests in an experimental design is statistically unacceptable; performing of a meta-analysis of multiple meta-analysis - a 'cumulative' meta-analysis - is equally statistically unacceptable. In both instances, the operation yields increased risk of Type I and Type II error, and loss of statistical power.

## The Bayesian Paradigm of "Additive" Meta-Analysis

The recommended statistical approach when performing a secondary- or tertiary-level meta-analysis is the Bayesian model. The Bayesian approach, originally proposed by Rev. Thomas Bayes (1701 - 1761) [42], estimates the progressive rapprochement of the accumulated observation to a true and complete representation of the absolute. It seeks to incorporate new findings with previous observations ("priors") to obtain an improved approximation of the population. It updates the probability estimate for a hypothesis as additional evidence is learned. This is opposed to the traditional frequentist probabilistic approach that does not promote the updating of information, and views parameters as fixed.

Bayesian reasoning stipulates that the knowledge of a whole is attained by means of adding new sets of observations to existing and previously observed ones (i.e., "priors"). It describes procedures for statistical inference in which the prior distribution is estimated from the data. The objectivist view of Bayesian inference rests on the rules and requirements of logic, rationality and consistency, but the subjectivist view seeks to quantify personal beliefs, and resulting inferences. In brief, Bayesian statistics includes a class of computational methods, which bypass the evaluation of the likelihood function, and rest on assumptions that can weaken the inferences [7, 43]

Bayesian interpretation of probability aims at evaluating the probability of a hypothesis based on some prior probability, which is then updated in the light of new, relevant data. It states that the odds of event X to event Y, before (prior to) and after (posterior to) conditioning on another event Z. The odds on X to event Y represent the ratio of the probabilities of the two events. The prior odds refer to the ratio of the unconditional or prior probabilities; the posterior odds are the ratio of conditional or posterior probabilities given the event Z. The relationship is expressed in terms of the likelihood ratio or Bayes factor, the ratio of the conditional probabilities of the event given that is the case or that is the case, respectively.

Bayes' rule of probability states that posterior odds equal prior odds corrected by the Bayes factor. In the Bayesian model, marginalized variables are parameters for a certain test (e.g., comparison), and the remaining variables confer the identity of the model itself [43]. That is, it provides an adequate approximation to the fully Bayesian treatment of a hierarchical model wherein the parameters at the highest level of the hierarchy are set to their most likely values, instead of being integrated out.

Bayesian statistics may provide precisely the theoretical framework that is needed to conceptualize how a meta-analysis in comparative effectiveness research, and specifically in the secondary- and tertiary-level meta-analytical models outlined above, particularly when dealing with the complex issue of individual patient data analysis and meta-analysis [7, 8, 11, 18, 44]. Specific advantages of Bayesian meta-analysis, and specifically a hierarchical Bayesian random-effects model, over the probabilistic approach include full allowance for all parameter of uncertainty in the model, inclusion of all other pertinent information that a probabilistic model would typically exclude, and expansion of the model to accommodate frequently occurring confounding variables [45].

In closing, timely and critical research has produced notable new developments in Bayesian meta-analysis. Software programs are now available to perform Bayesian inference (e.g., Using Gibbs Sampling [BUGS] coupled with the Markov Chain Monte-carlo [MCM]- R package; or the Gibbs sampler programmed in WinBUGS software [version 1.4, MRC Biostatistics Unit, Cambridge, UK]). These recent advancements undoubtedly ensure the dissemination of the Bayesian paradigm of secondary and tertiary-level meta-analysis in complex systematic reviews, and contribute to further advancing comparative effectiveness research, and more generally translational science in healthcare.

## Figures and Tables

**Table 1:** PRISMA Checklist [37]

Section/topic	Item	Checklist item
Title	1	Identify the report as a systematic review, meta-analysis, or both
Structured summary	2	Provide a structured summary including, as applicable, background, objectives, data sources, study eligibility criteria, participants, interventions, study appraisal and synthesis methods, results, limitations, conclusions and implications of key findings, systematic review registration number
Rationale	3	Describe the rationale for the review in the context of what is already known
Objectives	4	Provide an explicit statement of questions being addressed with reference to participants, interventions, comparisons, outcomes, and study design (PICOTS)
Protocol and registration	5	Indicate if a review protocol exists, if and where it can be accessed (such as web address), and, if available, provide registration information including registration number
Eligibility criteria	6	Specify study characteristics (such as PICOTS, length of follow-up) and report characteristics (such as years considered, language, publication status) used as criteria for eligibility, giving rationale
Information sources	7	Describe all information sources (such as databases with dates of coverage, contact with study authors to identify additional studies) in the search and date last searched
Search	8	Present full electronic search strategy for at least one database, including any limits used, such that it could be repeated
Study selection	9	State the process for selecting studies (that is, screening, eligibility, included in systematic review, and, if applicable, included in the meta-analysis)
Data collection process	10	Describe method of data extraction from reports (such as piloted forms, independently, in duplicate) and any processes for obtaining and confirming data from investigators
Data items	11	List and define all variables for which data were sought (such as PICOTS, funding sources) and any assumptions and simplifications made
Risk of bias in individual studies	12	Describe methods used for assessing risk of bias of individual studies (including specification of whether this was done at the study or outcome level), and how this information is to be used in any data synthesis
Summary measures	13	State the principal summary measures (such as risk ratio, difference in means).
Synthesis of results	14	Describe the methods of handling data and combining results of studies, if done, including measures of consistency (such as I2 statistic) for each meta-analysis
Risk of bias across studies	15	Specify any assessment of risk of bias that may affect the cumulative evidence (such as publication bias, selective reporting within studies)
Additional analyses	16	Describe methods of additional analyses (such as sensitivity or subgroup analyses, meta-regression), if done, indicating which were pre-specified
Study selection	17	Give numbers of studies screened, assessed for eligibility, and included in the review, with reasons for exclusions at each stage, ideally with a flow diagram
Study characteristics	18	For each study, present characteristics for which data were extracted (such as study size, PICOTS, follow-up period) and provide the citations
Risk of bias within studies	19	Present data on risk of bias of each study and, if available, any outcome-level assessment.
Results of individual studies	20	For all outcomes considered (benefits or harms), present for each study (a) simple summary data for each intervention group and (b) effect estimates and confidence intervals, ideally with a forest plot
Synthesis of results	21	Present results of each meta-analysis done, including confidence intervals and measures of consistency
Risk of bias across studies	22	Present results of any assessment of risk of bias across studies (cf. 15)
Additional analysis	23	Give results of additional analyses, if done (such as sensitivity or subgroup analyses, meta-regression)
Summary of evidence	24	Summarize the main findings including the strength of evidence for each main outcome; consider their relevance to key groups (such as health care providers, users, and policy makers)
Limitations	25	Discuss limitations at study and outcome level (such as risk of bias), and at review level (such as incomplete retrieval of identified research, reporting bias)
Conclusions	26	Provide a general interpretation of the results in the context of other evidence, and implications for future research
Funding	27	Describe sources of funding for the systematic review and other support (such as supply of data) and role of funders for the systematic review
